# Understanding healthcare autonomy among adolescents and young adults in the United States: a scoping review

**DOI:** 10.3389/frhs.2025.1720972

**Published:** 2026-01-12

**Authors:** Kelly L. Wilson, Sara Flores, Blessing O. Apata, Samia Tasnim, Whitney R. Garney, Kobi V. Ajayi

**Affiliations:** 1 College of Nursing, Texas A&M University, Bryan, TX, United States; 2 Center for Community Health and Aging, Department of Health Behavior, School of Public Health, Texas A&M University, College Station, TX, United States; 3 Department of Health Behavior, School of Public Health, Texas A&M University, College Station, TX, United States

**Keywords:** adolescent, autonomous care, decision-making, healthcare access, young adults

## Abstract

**Purpose:**

Maps out the evidence on AYA's autonomy and decision-making in healthcare settings in the United States to provide a comprehensive and synergistic understanding of the barriers, facilitators, and other salient factors that influence autonomous decision-making.

**Methods:**

This study followed the PRISMA and scoping review methodological frameworks. An electronic database search was performed using Boolean terms based on inclusion/exclusion criteria. Included studies were analyzed using narrative synthesis and thematic analysis techniques.

**Results:**

The final review comprised 31 studies. Half (*n* = 16; 52%) focused on adolescent autonomy in specialized care, a third focused on sexual and reproductive healthcare (*n* = 8, 25%), and the remaining studies focused on general healthcare (*n* = 6; 19%). Most studies defined autonomy as a primary influence in healthcare decision-making (*n* = 24; 77%). Other conceptual definitions focused on reproductive decision-making and control (*n* = 5; 16%) or independent functioning (*n* = 3; 9%). The literature discussed various barriers and facilitators to AYAs’ sense of autonomy.

**Conclusions:**

Studies regarding AYA autonomy have historically focused on specific patient populations in specialized healthcare areas. Researchers and practitioners can work towards creating tools to inform and assess interventions to support AYA autonomy in healthcare settings, including programs to improve care for youth.

## 1 Introduction

Approximately 42 million adolescents aged 10–19 constitute 12.8% of the United States (US) population (1) and the transition from this key demographic from adolescence to adulthood is characterized by emerging independence and autonomy (2). In general, autonomy refers to self-governance or independence; however, personal autonomy can be divided into four dimensions: `1) cognition—the expression of an individual's viewpoint and decision-making without external influence; 2) emotion—the process of individualization; 3) behavior—the ability to take responsibility for one's actions and making decisions for themselves; and 4) moral values—adhering to a set of moral values despite the situation or peer pressure (2–7).

In the healthcare setting, autonomous care may be used interchangeably with patient autonomy, which allows patients to make informed decision about their health that align with their values and preferences without undue coercion or influence (2–4). Autonomous care for adolescents transcends undue influence from health systems to policy and caregiver influences (3–7). As a result, adolescent and young adult (AYA) autonomy has various definitions depending on the setting.

AYA autonomy in the healthcare setting is often defined by legislation, which often differs between states (8). For example, in Texas-based healthcare systems, individuals younger than 18 are considered minors. Minors in Texas can consent to healthcare in certain situations and are allowed specific services without parental knowledge and consent. However, prescription birth control and other adolescent sexual and reproductive healthcare services require parental consent or notification (9). In contrast, minors in California have the right to access birth control (hormonal and non-hormonal methods), abortions, and STI/HIV testing and treatment without parental consent (10). These legislative complexities create a chasm in how healthcare is delivered by healthcare providers (HCPs) and consumed by adolescents.

HCPs are responsible for interpreting complex legislation regarding consent in adolescent healthcare, which can be challenging and restrict services. Many HCPs need more training on the topic, making them cautious to avoid any problems related to this issues (11). When unsure whether it is legal to accept adolescent consent over parental opposition or vice-versa, HCPs may concede decision-making control to parents by default (12). This tendency to default to the conservative position is influenced, in part, by HCPs believing parents hold their children's best interests at heart as they dominate the decision-making process (13).

Unclear policies and procedures in the healthcare setting regarding confidentiality also limit adolescent autonomy (8). Adolescents tend to be less active in discussion and healthcare decision-making during triangulated encounters between themselves, their parent(s), and HCPs (14). Adolescent involvement is further reduced when both parents are involved (15). This raises concerns due to well-known discrepancies between children's health self-reports and parent reports (16). Moreover, many minors are concerned their healthcare choices may be disclosed to their parents/guardians as supported in studies that show confidentiality concerns may prevent adolescents from seeking healthcare (8, 17). The impact of these concerns is much higher regarding potentially sensitive health services such as STI testing or mental health assessments (18, 19). Studies indicate adolescents with confidentiality concerns are less likely to disclose health-related information to their healthcare providers (HCPs), especially regarding issues related to mental health, substance use, and sexual behavior which may contribute to overall negative health outcomes (17).

Adolescent autonomy is a critical aspect of receiving comprehensive healthcare. It is argued that respecting adolescents' autonomy can promote their cognitive capacity to make sound healthcare decisions (20). Globally, it is regarded that children are capable of forming their views have the right to express them freely in all matters affecting them, with their age and maturity considered (21). This statement supports adolescents' right to consent to treatment and confidentiality in healthcare systems. The World Health Organization (WHO) recently created a tool based on principles of patient-centered care to assist assessment and support of adolescents' capacity for autonomous decision-making in healthcare settings (22). Notably, no formal guidelines regarding adolescent autonomy in healthcare were available before this tool was created. Increased understanding of teenage autonomy and healthcare decision-making among adolescents and young adults is imperative to inform standard practice and assess the new tool by WHO. This study aims to map out the evidence on AYA's autonomy and decision-making in healthcare settings in the US to provide a comprehensive and synergistic understanding of the barriers, facilitators, and other salient factors that influence autonomous decision-making.

## 2 Method

This study employed Colquhoun and colleagues' enhanced scoping review methodological framework, which outlines key processes when conducting a scoping review (23). The first stage involved linking and clarifying the purpose and research questions through deliberation and empirical reasoning, which led to identifying the relevant articles for inclusion in this study. Next, an iterative approach was used to select and extract the relevant data from the chosen articles. Lastly, a data analysis was performed using numerical and qualitative analysis to summarize and report results. A scoping review was appropriate for this study because it helps researchers understand the breadth and scope of adolescent autonomy, which remains an important yet understudied area (23). Moreover, a Scoping review methodology is ideal because it allows us to map and understand the existing literature on adolescent autonomy, identify knowledge gaps, and help map direction for future research, policy, and programs.

### 2.1 Search strategy

**Figure 1:**
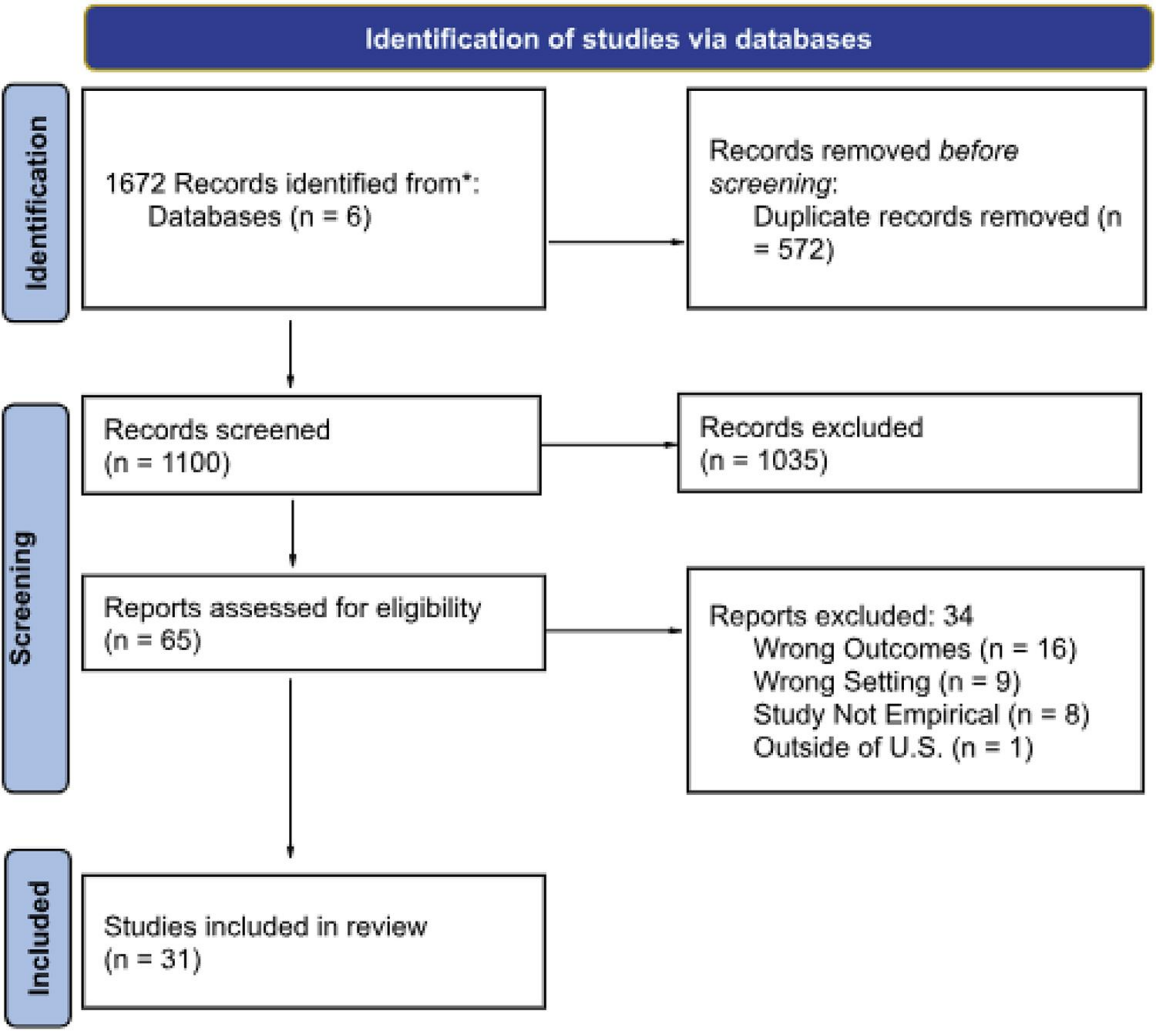
PRISMA flow diagram.

A comprehensive electronic database search of peer-reviewed literature was performed using six databases: Child Development & Adolescent Studies, CINAHL Complete, MEDLINE, Web of Science, PubMed, and PsychInfo from 2000 through March 2022. An updated search was conducted to include articles published from 2022 to 2025. Considering gaps in the literature, we included articles published in the last 25 years to help us provide rich data on existing literature. A Boolean search strategy with the “AND” and “OR” commands was used to extract relevant articles based on the following keywords in different combinations: (adolescent* or teen* or young adults or youth) AND (clinical decision support system or clinical decision making or decision-making) AND (personal autonomy or autonomy* or support for autonomy) AND (United States or America or USA or U.S. or United States of America or U.S.A.). A thorough hand search of each selected article's bibliography was conducted to identify other articles relevant to this study. The search outcomes were reported using the Preferred Reporting Items for Systematic Reviews and Meta-Analyses (PRISMA) guidelines, as seen in Figure 1.

### 2.2 Inclusion and exclusion criteria

Articles were required to be published in English and studies were conducted in the United States to be included in this study. We included qualitative, quantitative, and review studies reporting AYA healthcare autonomy in the United States. Based on existing literature, we defined AYAs as individuals between the ages of 10 to 31 years (24). For articles that included AYAs older than 31, only those with at least 50% of AYAs were included in this study. Additionally, we included studies conducted in various healthcare settings such as community centers, hospitals, or other places where AYAs receive health care. Similarly, studies focused on interventions, policy, or research-based (i.e., observational) were included. Articles were excluded if they reported autonomy outside the healthcare domain (e.g., autonomy in the education sector). Although we included research-based articles, we excluded randomized controlled trials because we were focused on autonomy within the context of healthcare utilization and AYA's interaction with providers while seeking care vs. the AYA's autonomy when participating in research not involving utilizing or interacting with a provider.

### 2.3 Data abstraction and analysis

A three-phase data extraction process was performed. Two reviewers independently screened the title and abstract, full-text, and then extracted data points into a matrix (25). Data extracted included, but was not limited to, study design, study demographic characteristics, study setting (i.e., hospital, school-based settings), health condition (i.e., cancer), the conceptualization of autonomy (i.e., how autonomy was defined), tools used to measure autonomy, and study findings. We also collected data on the barriers/facilitators associated with AYA's autonomous care. Next, the data were synthesized, narratively coded, and thematically analyzed to identify patterns, salient information, and themes related to AYA's autonomous care. Discrepancies were resolved through discussion and a third reviewer until an agreement and consensus were reached.

## 3 Results

### 3.1 Study characteristics

We included 31 studies in the final review and most studies (*n* = 24; 77%) included only adolescents and young adults (AYAs) (26–48). Other studies included adolescent-caregiver dyads or healthcare providers (49–56). AYA participants represented various subpopulations, such as those living with chronic conditions (e.g., cancer, type 1 diabetes mellitus, cystic fibrosis, or inflammatory bowel disease; *n* = 13; 42%) (30, 32, 33, 35, 40, 41, 44, 47, 49, 51, 52, 57), young women who have been commercially sexually exploited (*n* = 3; 9%) (26, 28, 39), a parous adolescent (*n* = 2; 3%) (36), and an adolescent in the juvenile justice system (*n* = 1; 3%) (37). Other study populations were broader in scope, including young women (*n* = 5; 16%) (29, 31, 40, 42, 53), undergraduate students (*n* = 2; 6%) (33), or adolescents and young adults in general (*n* = 5; 16%) (43, 48, 50, 54, 56). More than half (*n* = 16; 52%) of the studies focused on adolescent autonomy in specialized care (e.g., oncology, endocrinology, gastroenterology, behavioral health, and plastic surgery) (30, 32, 33, 35, 38–41, 43, 44, 47, 49, 51, 52, 56, 57). One-third of the studies focused on sexual and reproductive healthcare (*n* = 8; 25%) (29, 31, 36, 37, 42, 46, 50, 53), while the remaining focused on general healthcare (*n* = 6; 19%) (26, 28, 34, 45, 48, 54). Only one of the studies involved intervention research testing tools developed for AYA (49). Table 1 provides details regarding study characteristics.

**Table 1:** Characteristics of the included studies (*N* = 31).

Author/Publication year	Study sample/size	Study setting	Study dimensions	Study objective	Main findings
Barnert et al. (2019) (28)	Purposive sampling of commercially sexually exploited (CSE) women Ages 13–21 (final sample 15–19) (*n* = 21)	Community-based organization Residential treatment facilities (2) Juvenile court-based program	Research study; CSE young women receiving services from partnering organizations.	To understand the perspectives of commercially sexually exploited (CSE) young women regarding their healthcare needs, access, and utilization patterns.	CSE young women face significant health risks and substantial internal and external barriers to accessing healthcare. Their healthcare needs, barriers to care, and utilization patterns directly relate to their experiences of commercial sexual exploitation. CSE young women's utilization, engagement, and attitudes toward care were associated with the concept of fierce autonomy, which explained their desire for self-determination in their healthcare decision-making including access to care.
Biggs et al. (2019) (29)	Racial/ethnic minority adolescents aged 15–25 years (*n* = 22)	Youth-serving clinics	Research study; study participants were patients seeking contraceptive services from the youth-serving clinics	To understand young women's counseling experiences when accessing emergency contraceptives at family planning specialty clinics while gaining insights into what women appreciate and disliked.	Young women prefer contraceptive counseling that is supportive of their autonomous decision-making and offers guidance while addressing their preferences.
Brinkman et al. (2012) (30)	Adolescents with ADHD aged 13–18 years (*n* = 44)	Community-based pediatric practices	Research study; study participants were patients who had received ADHD care from the community-based pediatric setting	To gain a detailed understanding of how adolescents with attention deficit hyperactivity disorders contribute to medication treatment decisions.	Adolescents described their involvement in discussions and decision-making with their parents and doctors as inadequate. However, adolescents assumed increased responsibility for managing medication as they matured and developed insight into the functional impact of ADHD and medication on their lives.
Chang et al. (2018) (50)	Adolescents 14–17 years and a parent (*N* = 262) Qualitative analyses were conducted for those who agreed that they were offered and started the HPV vaccine series (*n* = 109)	Adolescent medicine clinics of 2 large urban medical centers	Research study; study participants were recruited from the adolescent medicine clinics who were offered and started the HPV vaccine	To examine how adolescent-parent dyads describe decision-making regarding initiation of the human papillomavirus (HPV) vaccine series, specifically who they viewed as making the final decision.	Most adolescents and parents described a similar account about when they were offered the HPV vaccine, although the interpretation of the event in terms of the decision-maker might have differed. More than half of adolescents and parents individually mentioned the health care provider in their description of the HPV vaccine decision-making process even though they were not queried about the role of the provider. About 57% of the dyads did not agree on who made the decision to start the vaccine series.
Dalessandro et al. (2022) (31)	Emerging adult women aged 18–24 years (*n* = 2594)	4 health centers	Research study;	To investigate how one potential indicator of reproductive autonomy—feelings of control over pregnancy—may relate to structural, relational, and individual factors in emerging adults’ (age 18–24) lives.	Most participants (86%) agreed with the statement “I feel that I have control over whether or not I get pregnant,” while the remainder were neutral or disagreed. Participants reporting poverty-level incomes and previous unwanted pregnancies were more likely to describe “neutral” feelings of control.
Darabos et al. (2021) (51)	AYA cancer survivor/caregiver dyads aged 15–29 years (*n* = 11) Caregivers of adolescents (*n* = 11) Oncology providers (*n* = 8)	Cancer center at a pediatric hospital	Research study; adolescents with a history of cancer and receiving treatment, their caregivers, and providers from the children's hospital participated in this study.	To provide novel insights into decisional processes that can facilitate optimal engagement in decision-making, ultimately informing potential targets of intervention to support decision-making among adolescent and young adults.	As expected, AYA are highly engaged in decision-making, weighing options presented by oncology providers and making decisions together with their caregivers. AYA mentioned jointly engaging in decisions with their caregivers and oncology providers (i.e., collaborative decision-making) for treatment-related decisions.
Godoy et al. (2020) (26)	Purposive sampling of commercially sexually exploited women ages 15–19 years (*n* = 21)	Juvenile specialty court, foster care group home agencies, and local service providers serving youths who identify as commercially sexually exploited (CSE).	Research study; CSE young women receiving services from partnering organizations.	To understand the narratives and views of individuals affected by CSE on their bodies, health, and motivations to seek health care treatment.	CSE women have a high drive for autonomy over their bodies and health-related issues, which is influenced by their unique backgrounds, including the lack of control over their own bodies and decisions and high health care needs.
Hanna & Guthrie, (2003) (32)	Adolescents with T1 diabetes ages 11–18 years (*n* = 34)	Diabetes specialty clinics	Research study; participants were recruited from diabetes specialty clinics	To examine independent functioning and decision making for stages of early, middle, and late adolescence, and the relationships among independent functioning and decision making for daily management, typical adolescent issues, and metabolic control among adolescents with type 1 diabetes.	Overall, adolescents were somewhat independent in functioning and decision-making for both daily diabetes management and adolescent activities. However, adolescents had lower scores of independent functioning and decision-making for nondaily diabetes management. Compared to older adolescents younger adolescents were less independent in daily diabetes management functioning.
Miano et al, (2020) (33)	AYA patients who received treatment in pediatric hematology/oncology clinics ages 14–25 years (*n* = 46)	Pediatric hematology/oncology clinics at an academic children's hospital	Research study; participants were recruited from the participating children's hospital	To describe decisional control preferences of a previously understudied population and explore factors that may impact desired levels of decisional control for AYAs with cancer and other complex medical conditions.	39% of patients preferred an active collaborative role, where the patient preferred to make the final decision with input from the provider. 34% of patients prefer a shared decision-making role wherein the decision is jointly made between the patient and provider. Compared to non-oncology patients, oncology patients appeared to prefer a more passive role. As time since diagnosis progressed, patients tended to prefer a more active level of decisional control. A strong correlation between self-efficacy for decision-making and self-regulatory skills, perceived autonomy, social support, and shared decisions.
Miller et al. (2007) (52)	Adolescents aged 11–17 years with type 1 diabetes and their mother dyads (*n* = 82)	Clinic setting	Research study	To document the relationship between discrepancies in mother and adolescent perceptions of diabetes-related decision-making autonomy, diabetes-related conflict, and regimen adherence.	Discrepancies between mother and adolescent perceptions of decision-making autonomy were related to a greater maternal report of diabetes-related conflict. Particularly, when adolescents reported that they were more in charge of decisions than reported by their mothers, mothers reported more conflict. Discrepancies between mother and adolescent perceptions of decision-making autonomy were not related to regimen adherence.
Nicoteri & Arnold (2005) (34)	Young adults aged 18–23 year (*n* = 8)	University	Research study	To illuminate the process of the development of health care–seeking behaviors in traditional-age undergraduate college students by asking how students develop independent or autonomous health care-seeking behaviors.	Students primarily make their own healthcare decision but would frequently defer to parents. The findings indicate these traditional-age undergraduate college students view themselves as independent but still rely on parental supervision of health care. Involvement in healthcare decisions was largely a function of how involved the parents were in their children's lives.
Pindar et al. (2020) (53)	Adolescents and young adults aged 14–21 years (*n* = 89)	Outpatient pediatric clinic	Research study; participants were patients from the outpatient pediatric clinic	To examine the association between reproductive autonomy and adolescent receptivity toward long-acting reversible contraceptive (LARC) methods	Overall, adolescents demonstrated reproductive autonomy with only less than one-fifth of the sample population reporting that their reproductive decision was made entirely by some other than themselves. Additionally, adolescents felt that they could communicate with either their partner or parent about sex and pregnancy.
Plevinsky et al. (2015) (35)	Young adults diagnosed with inflammatory bowel disease (IBD) aged 18–30 years (*n* = 29)	Children's hospital center	Research study; participants were former patients at a children's hospital center for inflammatory bowel disease	To (1) explore the transition experience of young adults with IBD; (2) understand the impact of the pediatric experience and patient-provider relationship; and (3) identify the contribution of patient characteristics (i.e., diagnosis age, illness severity, parent involvement) to the adult experience and patient-provider relationship.	Positive themes regarding adult providers included independence, autonomy and trust, while negative themes included initial discomfort and confusing logistics. The earlier the diagnosis age, the less involved in medical decisions they were as an adult. Those who had a more positive experience with their adult providers were more likely to endorse collaborative medical decision-making.
Ranganathan et al. (2020) (56)	Children and adolescents aged 6–18 years and their caregivers (*n* = 100)	Plastic surgery clinic	Research study; patients presenting at a plastic surgery clinic	To define the preferred approach to decision-making for pediatric patients and their parents in a plastic surgery setting, and identify a model of shared-decision-making that balances preferences between the various stakeholders involved in surgical care.	Overall, 40% of children and 67% of caregivers preferred the option of completely shared decision-making between the patient, caregiver, and surgeon. 0The minority of children (16%) preferred the surgeon to be the main source of decision-making, while almost 20% of children desired complete autonomy.
Roque et al. (2022) (36)	Parous female adolescents 16–19 years old (*n* = 12)	Medical center	Research study; patients attending a labor and delivery unit at a medical center	To better understand the influences and factors surrounding contraceptive decision-making in adolescents following the index delivery	Adolescents’ contraceptive decision-making was influenced by their friends, providers, and family members, but in particular, mothers played a key role. Contraceptive decision-making was often collaborative, with adolescents valuing the opinions of their mothers, sisters, and friends. Post-delivery adolescents had more autonomy to make decisions regarding their contraception choices.
Squitieri et al. (2013) (57)	Adolescents with neonatal brachial plexus palsy aged 10–17 years and their parents (*n* = 18)	Patient network	Research study; patients needing surgery, Botox, or other types of care related to neonatal brachial plexus palsy through patients’ network.	To explore and describe the medical decision-making process among children and adolescents with neonatal brachial plexus palsy from the patient and family perspectives.	Adolescents largely based their medical decision-making on individual preferences, but their parents were heavily influenced by system-dependent factors, such as the Internet, information obtained from other parents, and logistical coordination of care. Patient-dependent factors, such as adolescent autonomy and individual expectations/treatment desires, are characteristics unique to each adolescent and generally unable to be influenced by physician interactions or delivery of care.
Ti et al. (2019) (37)	Incarcerated girls aged 13–18 years (*n* = 22)	Juvenile detention center	Research study; participants were recruited from the juvenile detention center	To use a framework of PCC to describe the experiences and preferences of incarcerated girls with receiving family planning care within a juvenile detention center	Incarceration limited increased stigma and limited autonomy leading to tension toward receipt of reproductive health care. Participants’ desire for autonomy contributed to concerns around FP care. Despite this, most desired access to FP care while incarcerated. Ironically, being incarcerated led to an opportunity for new autonomy and self-reliance, because for some this was the first time seeking care independent of family and friends.
Weaver et al. (2015) (38)	AYA Cancer Survivors aged 12–18 year (*n* = 40)	Private inpatient or outpatient rooms in clinics.	Research study; participants receiving care from the recruiting clinics	To investigate the medical decision-making preferences of adolescent oncology patients and the parental and clinician behaviors that adolescents report to be supportive of their preferred level of decision-making involvement.	Adolescents indicated a spectrum of preferred decisional roles, with the most common being an actively involved role (*n* = 26 or 65%), although a shared decision-making approach was still valued. There was no statistically significant difference in the preferred decisional role with respect to demographic or medical characteristics, including the relapse status, although adolescents who preferred autonomous interview settings were more likely to prefer active decisional roles. Adolescents recognized that situational and social contexts might shift their preferred level of involvement in medical decisions. Although adolescents wanted to be involved in decisions, they also expressed an appreciation of family insight, parental presence, and clinician guidance.
Corona et al. (2022) (39)	Purposive sampling of AYA with Differences of Sex Development (DSD) Aged 14–28 years (median 17 years) (*n* = 8)	Multidisciplinary DSD clinic	Research study, participants were enrolled in a DSD-specific gonadal tissue cryopreservation (GTC) protocol by 4 multidisciplinary hospitals	To examine the fertility-related attitudes and experiences of AYA with DSD to inform future care needs	AYA expressed openness regarding fertility preservation options, desired for full disclosure of information, acknowledged age-related progression to autonomy for decision making and willingness to take more responsibility with as they get older
Daraiseh et al. (2022) (49)	Purposive sampling of AYA with Ulcerative Colitis (UC) 14 AYA, 6 HC worker, 4 designer, a social worker and a human factor researcher Aged 15–19 years (median 16 years) (*n* = 14)	Pediatric inflammatory bowel disease (IBD) center in the hospital	Research study, evaluating a app co-designed with AYA to help with treatment related decision making	To identify the key components and design features of a decision tool for AYA patients with UC and optimize the tool for usability, acceptability, and decision-related outcomes using human factor usability	AYA participants found the app to be organized, and the information provided were streamlined and easily accessible. Medication and nutrition trackers were considered as a positive feature. They expressed desires to incorporate the app with the portal to communicate with their health care provider.
Delgado et al. (2023) (54)	Purposive sampling of HCW (*n* = 21) and their adolescent children (*n* = 17)	Integrated health system in Southern California	Research study, exploring parents and their adolescents’ behavior regarding Covid-19 vaccine	To explore the perceptions of healthcare worker parents and their adolescents’ adolescent self-consent to COVID-19 vaccination by applying the concept of positive deviance among those already vaccinate	Decision related covid 19 vaccines were made by parent in majority of cases. Both parent and AYAs mentioned the influence of environment (such as school) and peers on this decision making. Regarding vaccine self-consent, many parents were supported and considered it as part of the medical autonomy where are some parents only supported this for older teens. AYAs supported self-consent, some mentioned it could be useful for children of anti vax parents a few AYA preferred parental decision over self-consent in case of vaccines citing their parents’ in dept knowledge and experience.
Golden et al. (2022) (55)	238 medical decisions recorded across the 4 popular TV shows of their time period was reviewed	NA	Research Study, reviews depiction of medical decision making in TV shows	To aimed to investigate the manner in which four selected TV dramas have depicted the pediatric patient's degree of participation in shared decision making.	Statistically significant (*p* = 0.050213) increase in pediatric decision making was observed over time, with 44% patients in ER (1994) vs. 66.7% patients in The Good Doctor (2017) being involved in decision making.
Moskop & Derse (2024) (40)	1 AYA	Emergency care	Case study, assessing moral dilemma for autonomy, harm, responsibility, and justice for a minor	Examine the ethical challenges posed by the prolonged boarding of medically stable but socially vulnerable AYA, through the case of a 17-year-old in emergency departments, focusing on the principles of autonomy, nonmaleficence, and distributive justice	This case study of a 17-year-old diabetic patient explored the ethical concerns for caring a medically stable but socially vulnerable AYA in emergency department. The ethical review board reported that she displays limited decision-making capacity evident by inconsistencies diabetic care in the past. The board deemed that her refusal to remain in the hospital should be honored if she seemed capable. Although releasing her from the hospital without a safe destination can worsen her health, extended stays against her wishes will also be harmful. Keeping her will also be harmful for her care givers due to her hostile behavior towards them and this also consume scarce medical resource delaying care for people with urgent needs. Considering the ethical issues the board approved her release from the hospital despite no safe destination for her to return.
Pyke-Grimm et al. (2022) (41)	Purposive sampling of AYA with cancer diagnosis Aged 15–20 years (mean 17.3 years) (*n* = 16)	Pediatric oncology hospitals	Research Study, assessing the impact of cancer treatment on everyday decision making	To explore involvement of AYAs with cancer in day-to-day decisions affected by their cancer and treatment.	Four key themes identified were 1) mental mindsets 2) self-care practices 3) self-advocacy and 4) navigating relationships. The mentioned that accepting their diagnosis and the mind set of fighting the disease helped them to continue their treatment. Self-care practices like ensuring proper hydration, caring central line, self-administration of drugs for chemo or fertility preservation were also helpful. Self-advocacy for preventing complications and behaviors that facilitate treatment was also mentioned. Lastly, they mentioned the changes in their relationship with friends and family due to these treatments and shift in reliance on peers and social media for support.
Rao et al. (2023) (42)	Sexually-active individuals assigned female at birth Aged 16–29 years (mean 21 years) (*n* = 30)	Reproductive health clinic	Research study, aimed to explore the meaning of agency in seeking contraceptive care	To explore what agency means to patients seeking contraceptive care to inform the development of a validated measure of this construct	Three main themes identified were: clear communication and realistic expectations, freedom from pressure or coercion, and a non-judgmental clinical environment. Effective communication involved the use of accessible language, setting accurate expectations especially about side effects and building provider trust with time. Many valued leading their own decisions and sometimes changed providers for greater agency. Experiences of coercion often had lasting negative emotional effects, while non-judgmental care fostered comfort and empowered decision-making.
Rea et al. (2023) (43)	Convenience sampling of AYA and one of their parents Mean age for AYA = 15 years *n* = 14 AYA, *n* = 20 parents	Pediatric Primary care clinic	Research study aimed to explore issues of privacy in telehealth approaches for AYA	To explore how telehealth contributes to and impede adolescent autonomy fostered through the organization of privacy.	Privacy in telehealth care was deemed crucial for adolescent development. Adolescents felt more emotionally secure and open to discussing sensitive issues when they had alone time with healthcare providers (HCPs). However, some adolescents appreciated having parents present for reassurance and support, which contributed to their emotional safety and learning. Parents had mixed views on privacy; many did not consider their children as autonomous individuals who needed privacy with HCPs, while others supported HCPs meeting alone with their kids.
Sutherland-Foggio et al. (2024) (44)	AYA with advanced cancer diagnosis Aged 10–23 years (mean 15.37 years) (*n* = 41)	Pediatric cancer care hospital	Research study, qualitative and quantitative representation of AYA decision making for advanced cancer treatment	To describe perceptions of AYA in decision making, frequency and desire for involvement in care, to examine factors associated with involvement in care	Overall AYA were satisfied with level of decision making and had little to no desire for changing level of changes in their involvement. There was no association with time since diagnosis. Both qualitative and quantitative findings reveal that older AYA had a greater desire for involvement and autonomy reflected by their higher frequency of involvement. Majority of the participants felt their decisions were made collaboratively and trusted their family to make decision for them.
Valente et al. (2022) (45)	Young cisgender men who have sex with men (YMSM) with negative HIV status or unknown status Aged 15–24 years (*n* = 737)	YMSM recruited online	Research Study,	To identify subgroups (latent classes) of YMSM based on their patterns of sexual health decision-making and healthcare access and examine how these patterns relate to preferences for different PrEP modalities.	Three decision-making classes identified were shared decision making (35%), provider lead (25%) and patient driven (41%). Having health insurance coverage, regular provider was associated with greater PrEP awareness, HIV testing and PrEP use. Daily oral PrEP was the preferred option, and shared decision makers were open to alternatives like injectables and implants. 31–57% experienced stigma and mistrust but it was not a predictor for class membership.
Wood et al. (2024) (46)	AYA with recent history of STIs Aged 13–19 years (median age 17.4 years) (*n* = 35)	Primary care and family planning clinic	Research study, assessing perspective of AYA with recent STI infection for prevention programs	To highlight the perspective of AYA with recent history with STI infection for novel STI prevention programs	Mental health was an upstream contextual factor influencing HIV/STI prevention attitudes, norms, and self-efficacy. They desired prevention counseling that allowed for decisional autonomy and individualized goal setting. Non-judgmental support affirming their ability to make decisions in line of their health goals and sexual orientations was sought. Negative social norms such as using condoms as indicators for distrust or infidelity that lower compliance with condom use. The need for training in effective communication about STI diagnosis with their partners citing their previous difficulties in sharing the information. Desired for detailed information about prevention of STIs in future. Many has positive experience with diagnosis and treatment but had to rely on internet about knowledge on prevention.
Woolley et al. (2023) (47)	AYA with Cystic Fibrosis (CF) Aged 12–20 years (mean 15.51 years (*n* = 39)	Pediatric CF care center	Research study, examining the attitude of AYA regarding communication with providers	To explore the attitudes of AYA with CF about communication with providers and factors improving communication	Participants expressed a desire to be actively engaged in discussions about their health-related decision-making with their providers and identified factors that improve and hinders development of autonomy
Xu et al. (2024) (48)	College going AYA Ages 18–24 years (mean age 20.15 years) (*n* = 30)	Participants from the HPV control study	Research study, assessing mechanisms increases uptake of HPV vaccination	Narrative exploration of HPV vaccination decision-making among racial/ethnically diverse young adults	Convenience was the key factor deciding about vaccination. Sense of adulthood increased accountability and heightened ability to make decisions. Access to preventative care and destigmatizing the norms against HPV vaccination were also mentioned as enabling mechanism.

### 3.2 Definitions and measures of autonomy

Most studies defined adolescent autonomy as a primary influence in healthcare decision-making (*n* = 24; 77%) (30–34, 36, 38–41, 44–57). Some conceptual definitions were more specific, focusing on reproductive decision-making and control (*n* = 5; 16%) (31, 36, 42, 46, 53) or independent functioning regarding medication management (*n* = 3; 9%) (30, 32, 52), privacy in telehealth care (43). Studies used various tools to assess adolescent autonomy. Majority of the included studies used author-created or modified tools to assess adolescent autonomy (*n* = 20; 65%) (26, 30, 31, 35–39, 41–43, 45, 47–50, 52, 54, 55, 57), only five studies (16%) relied on previously validated scale (32, 33, 44, 52, 53) but only a few reported tool validity (*n* = 8; 26%) (36–38, 44, 52, 53). The remaining studies (*n* = 7; 22%) did not directly measure adolescent autonomy (26, 28, 29, 34, 40, 51, 56). Table 2 shows a summary of the tools used to measure adolescent autonomy.

**Table 2:** Definition and assessment of autonomy utilized by the included studies (*N* = 31).

Author/Publication year	Conceptual definition of autonomy	Assessment tools/use	Validity of tools
Barnert et al. (2019) (28)	While the authors did not measure or operationalize autonomy, they conceptualized autonomy based on their findings called “fierce autonomy.” Fierce autonomy refers to the idea that CSE young women, who are often under the control of their trafficker, often tend to develop a resolute attachment to preserving their decision-making capacity. The framework conceptualizes how marginalized young women exhibit autonomy over their bodies, and particularly their healthcare decision-making.	NA	NA
Biggs et al. (2019) (29)	The authors did not define or conceptualize autonomy, instead, they assessed participants’ attitudes toward emergency contraceptive use as a proxy for their autonomous contraceptive decision-making.	NA	NA
Brinkman et al. (2012) (30)	The authors framed the research questions to elicit adolescents’ decision-making about the continued use of ADHD medications	Battery of interview questions on decision-making developed by authors	NA
Chang et al. (2018) (50)	Decision making	Interview questions on decision-making were developed by authors. “How did you make a decision about whether or not to receive the HPV vaccine series?”	NA
Dalessandro et al. (2022) (31)	"Reproductive autonomy or the ability to control pregnancy decisions, including pregnancy prevention, contraception, and the choice to continue a pregnancy."	Responses to the survey statement, “I feel that I have control over whether or not I get pregnant,” served as the dependent variable in the analysis. They analyzed whether participants agreed, disagreed, or were neutral in their response.	NA
Darabos et al. (2021) (51)	Decision making	Cognitive and emotional decision-making (CEDM) approach framework	NR
Godoy et al. (2020) (26)	The authors described the “fierce autonomy” model to elucidate CSEs agency related to decision-making related to their healthcare	semi-structured interview guide with three distinct sections, capturing the participants’ views on (a) health, (b) health care access, and (c) recommendations for improving access to care.	NA
Hanna & Guthrie, (2003) (32)	Behavioral autonomy, which is considered to include both independent functioning and decision making.	Independent functioning in daily and nondaily diabetes management checklists developed *a priori* from the Diabetes Family Responsibility Questionnaire and from the literature. Independent functioning in typical adolescent activities/rules checklist adapted from the adolescent decision-making scale and the issues checklist. Independent decision making in daily and nondaily diabetes management checklist. Independent decision making in typical adolescent activities/rules checklist.	Content validity of the independent functioning/decision making in daily and nondaily diabetes management checklist was achieved by a review of the checklist by a physician-researcher who is an expert in adolescence and diabetes.
Miano et al, (2020) (33)	Decisional control preferences	Decisional control preference was measured using the Control Preference Scale, Patient experience with SDM was measured using the three-item CollaboRATE tool, Self-efficacy for decision-making was measured using the Decision Self-Efficacy Scale, the Adolescent Self-Regulatory Inventory (ASRI) was used to measure patient self-regulatory skills, Perceived autonomy was measured by the Health Care Climate Questionnaire (HCCQ), and Social support was operationalized with the Multidimensional Scale of Perceived Social Support.	Empirically validated tools.
Miller et al. (2007) (52)	decision-making autonomy	Diabetes-Related Autonomy was measured using the Deciding About Diabetes Treatment Scale, Diabetes-Related Conflict was measured using the Conflict subscale of the Diabetes Responsibility and Conflict Scale, and Adherence to treatment regimen was measured using the Self-Care Inventory measures.	Empirically validated tools.
Nicoteri & Arnold (2005) (34)	Autonomous decision making skills	NA	NA
Pindar et al. (2020) (53)	Autonomy was measured using the reproductive autonomy decision-making subscales: Self-efficacy, decision-making, and communication Reproductive autonomy is defined as “having the power to decide about and control matters associated with contraceptive use, pregnancy, and childbearing” (Upadhyay et al., 2014).	Reproductive Autonomy Scale	Empirically validated tools.
Plevinsky et al. (2015) (35)	Autonomy was not measured	Developed the “Transition to Adult Care Questionnaire"	NR
Ranganathan et al. (2020) (56)	Decision-making preferences (main source of decision-making)	The authors simulated scenarios where the adolescents and their caregivers were given five different scenarios that both verbally and pictorially depicted different methods of decision-making ranging from completely autonomous to surgeon-determined	NA
Roque et al. (2022) (36)	Decision-making and choice	The authors developed an interview guide that explored sources of influence for contraceptive decisions, reproductive goals, and impact of pregnancy and delivery on goals and contraceptive choice	Validation was done by pilot-testing the interview questions with three participants
Squitieri et al. (2013) (57)	Decision-making: Ability and desire to independently communicate with a physician and influence the medical decision-making process.	Interview guide developed by authors on medical decision-making and quality of life	NR
Ti et al. (2019) (37)	The authors did not directly measure autonomy, but the interview guide asked questions about participant's Family Planning (FP) preferences.	Interviews covered 2 general topics: (1) past experiences with FP; and (2) preferences for FP services while incarcerated, with specific questions centered around domains of patient centered care.	Interview guides were piloted with nonincarcerated youth and modified on the basis of youth feedback. Individual interviews were conducted in person in private rooms at the clinic in the Juvenile Detention Center.
Weaver et al. (2015) (38)	Decision-making	The authors assessed decision-making using open-ended questions. Role preferences were converted into a predetermined Likert scale decisional preference score.	The interview guide was reviewed by a qualitative expert and pilot-tested by four adolescents.
Corona et al. (2022) (39)	Experience regarding decision making related to medical care	Interview guide was developed by the authors	NA
Daraiseh et al. (2022) (49)	Shared decision-making regarding treatment and lifestyle changes regarding UC management	Interview guide was developed by the authors	NA
Delgado et al. (2023) (54)	Self-consent for vaccines	Interview guide was developed by the authors	NA
Golden et al. (2022) (55)	Autonomy was defined as the ability to have options and having the opportunity to choose from them regarding treatment. Shared decision making was defined as situation where patients were informed about a potential medical decision and were able assert their own decision irrespective of parental or provider support	Interview guide was developed by the authors	NA
Moskop & Derse (2024) (40)	Decision making capacity of minors regarding lifesaving care	NA	NA
Pyke-Grimm et al. (2022) (41)	Shared decision was defined as a partnership between the patient and their providers for making treatment related decisions by informing the patients about the condition there by increasing their knowledge and reduce decisional conflicts	Interview guide was developed by the authors	NA
Rao et al. (2023) (42)	Contraceptive agency was defined as an individual's ability to make choices about contraception, including whether or not to use contraception and which method to use. They used *Kabeer* et al.*,* definition of agency as a woman's ability to make strategic life choices and considers communication, decision-making, and freedom from coercion, among other factors as their guide	Interview guide was developed by the authors	NA
Rea et al. (2023) (43)	Privacy was defined as the inherent right of patients, which includes confidentiality and respect for health care information	Interview guide was developed by the authors	NA
Sutherland-Foggio et al. (2024) (44)	Decision making	Decision making survey- developed by authors Autonomy and Information Seeking Preference Scale (AISPS) Family Roles Questionnaire (FRQ)	Decision making survey- tested for validity (internal consistency for intrinsic (*α* = .79) and extrinsic (α = .80) subscales) AISPS and FRQ are empirically validated tools
Valente et al. (2022) (45)	Decision making	Interview guide was developed by the authors	NA
Wood et al. (2024) (46)	Preference for STI prevention care	Interview guide was developed by the authors	NA
Woolley et al. (2023) (47)	Autonomy in clinical care	Interview guide was developed by the authors	NA
Xu et al. (2024) (48)	Decision making	Interview guide was developed by the authors	NA

NA, not applicable; NR, not reported.

### 3.3 Preferences for autonomy

AYAs showed a spectrum of preferred levels of involvement in healthcare decision-making, ranging from complete deference to complete autonomy. However, they overwhelmingly preferred (*n* = 15; 48%) active involvement characterized by shared decision-making with input from their healthcare providers and caregivers (29, 33, 36, 38–42, 44, 47, 51, 53, 54, 56, 58). In addition, AYAs' desire for autonomy appeared dynamic, shifting in different situational and social contexts. For example, young women who were commercially sexually exploited exhibited an amplified desire for autonomy in healthcare decision-making (26, 28). A study of AYA oncology patients showed a preference for more passive roles in their care compared to non-oncology patients (33). Notably, survivors' preferences for autonomy shifted to more actively involved roles as time passed since diagnosis. A different study focused on AYA oncology patients reported no significant difference in preferences for autonomy between those undergoing treatment for a new diagnosis and those undergoing treatment for a relapsed cancer (38). Age of patients also appeared to play a substantial role in preferences for autonomy, with younger children more likely to defer decisions or responsibilities to caregivers or healthcare providers compared to older adolescents and young adults (30, 32, 35, 56, 57).

### 3.4 Barriers and facilitators of autonomous care

#### 3.4.1 Barriers

The barriers for autonomous care are classified into individual, interpersonal and institutional and policy levels.

##### 3.4.1.1 Individual factors

At the individual level, being younger was the most common barrier reported. Several studies reported that younger AYAs were less likely to engage in health-related decision-making, be involved in their diet and self-management for diabetes, and inflammatory bowel disease (IBD) (32, 35, 39, 53, 54). Similarly, Corona et al., 2022 assessed fertility preservation-related decision-making among AYAs with cancer and found that they often did not perceive fertility as an important concern; consequently, they did not engage in any decision related to it (39). Lack of knowledge and experience in health care was another commonly cited barrier (41, 44, 45, 55). These studies mention that AYAs often have inadequate knowledge and understanding of their health conditions, hindering their care involvement. Pyke-Grim et al., 2022 reported that many AYAs have relatively less understanding of their diagnosis and experience in the health care system, in the early phase of treatments, and rely more on their parents and providers (41). Several psychological factors, such as anxiety and fear related to their diagnosis, also prevented their involvement in care. For example, Corona et al., 2022 reported that youth involved in their study often had limited participation due to fear and anxiety related to their health outcomes (39). Negative experience also played a role in shaping their involvement (26, 52). For example, Godoy et al. 2020 reported that previous experiences from exploitation, unstable family backgrounds, and involvement in the system of care hindered young women in their study from having control over their bodies, and by extension, their health care decisions (26).

##### 3.4.1.2 Interpersonal factors

Factors related to their relationship with parents, caregivers, and providers are included at the inter-personal or family level. Limited communication and shared decision making with parents and providers were common factors identified by the included studies (30, 45, 47, 52). For example, Brinkman et al., 2012 reported that limited involvement in decision-making and discussion between AYA, parents and providers led neglect of ADHD treatment (30).

Conflicting relationships with parents was another important factor influencing autonomous care (40, 47, 48, 52, 54). While conflicting relationships with parents, especially mothers hindered autonomous decision-making for contraception (36), many parents did not support the idea of self-consent for medical procedures such as vaccinating against COVID-19, thereby limiting their abilities to make independent health decisions (54).

Some studies found the AYA often trusted their family members and friends more than their providers, as a result they relied on their support network to make their medical decisions instead of being directly involved with their health care providers for making decisions. For example, Roque et al. 2022 reported that AYA relied on their mothers, sisters or other family members contraceptive-related decisions instead of their providers (36).

Peer influences from school-based social dynamics also emerged as another factor influencing AYA (48, 54). Both the studies reported that AYA included in their studies were influenced by their peers for vaccine related decision making, and positive social norms for vaccination increased likelihood of vaccine uptake.

##### 3.4.1.3 Community and institutional factors

Community and institutional level factors include larger systems and structures that shape their autonomy in health care. Institutional structure was one of the notable factors reported by the studies included (26, 37, 43, 45, 48). One of the studies mentioned that incarceration increased stigma for contraceptive care, limiting AYAs' autonomy for family planning, however it also instilled a sense of self-reliance by allowing them to make decisions independent of the influence of their family and friends (37). Location of care and the type of technologies used also played a role in shaping their experiences. Rea et al., 2023 explored privacy concerns related to telehealth care and reported, that parents often did not consider AYA as an autonomous individual requiring confidentiality while meeting their providers. AYA had mixed reactions, while some found the presence of a parent during tele visits reassuring, majority reported feeling more emotionally secure and open to discussing sensitive issues when they had alone time (43). Additionally, technology such as video calls also raised the issues of privacy (43).

Judgmental and dismissal attitude from providers also played an important role (42, 46). For example, Rao et al., 2023 found that AYA judgmental attitude for contraceptive related decisions, dismissing symptoms after contraceptive use and feeling pressured to use contraceptives compromised their sense of agency (42).

Ethical and legal considerations also emerged as potential barriers to autonomous care. Moskop and Derse, 2024 presented a case study involving an ethical dilemma in which a minor with minimal social support and limited capacity to self-manage diabetes was initially not released from care due to the risk of life-threatening complications (40). Although the minor in this case study was ultimately released, the ethical tensions for releasing minors in similar high-risk cases persist. Another study by Delgado et al., 2023 also reported that parents and healthcare providers hold mixed opinions about lowering the legal age of self-consenting for vaccination (54).

Lastly stigma and social norms was also a critical factor for AYA, studies assessing autonomy in sensitive issues such as HIV prevention (45), STI prevention (46) or HPV vaccine uptake (48) reported that stigma and social norms often constrained their ability to make informed and autonomous decisions.

#### 3.4.2 Facilitators

##### 3.4.2.1 Individual factors

Among the individual level facilitators, older age was mentioned by several studies (35, 44, 45, 56, 57). Older AYA demonstrated greater developmental maturity and communication skills which facilitated shared decision-making. For example, Squitieri et al., 2013 reported older age increases adolescents' ability to communicate independently with their providers while also increasing their medical decision-making agency (57). Knowledge and understanding of the disease and the treatment procedure also increased their autonomy in receiving care (39, 55). While internet increased their access to medical information (55), comprehensive understanding allowed for informed decision making (39).

Major life events (e.g., childbirth, commercial sexual exploitation, incarceration, etc.) appeared to relate to a sense of autonomy among AYAs (28, 36, 37). For example, parous adolescents exhibited an enhanced sense of reproductive autonomy after giving birth, relying more upon themselves to make decisions regarding contraception (36).

Self-reliance was another factor identified by the studies included (26, 35, 36, 41, 42). These studies reported that adolescents with higher self-reliance had greater autonomy in their care. Self-reliance increased progressively with age and duration of treatment. For example, Pyke-Grim et al., 2022 found that among the survivors of pediatric cancer, over the course of the treatment, adolescents developed skills for self-advocacy which allowed them to manage their treatment schedule, request for accommodations for family members or specific providers and were able to make collaborative decisions for their care (41).

##### 3.4.2.2 Interpersonal factors

Trust in family members and health care providers was the vital factor facilitating autonomous care for AYA (35, 44, 47). The study by Plevinsky et al., 2015 found that, building trusted relationship with providers increased independence and autonomy (35). Another study by Sutherland-Foggio et al., 2024 examined autonomous care among adolescents with advanced care and reported trust in family and health care providers allowed collaborative decision-making and increase in autonomy (44).

Patient centered care and shared decision-making between AYA, parents and providers was the most frequently cited facilitators by the studies included (29, 33, 35, 36, 38, 39, 41–43, 45, 49, 51, 52, 55, 57). These studies emphasized that institutional practices supporting patient centered care fostered a collaborative environment where AYA could better process health-related information, understand the roles of different providers, navigate the complex health care system and manage transitions between different types of care. This helped with increased self-efficacy and informed decision making. For example, Weaver et al., 2015 found reciprocal relations with clinicians allowed both patients and providers to understand the social and situational context, leading to shared decision-making (38). Corona et al., 2022 also reported that AYA valued access to various medical experts and comprehensive explanations of their treatment options, which facilitated understanding and supported informed choices (39). For tele-health care services, while certain technologies compromised their privacy, other types of technologies such as headphones, chat features facilitated establishing close connection with providers allowing for more autonomy (55).

## 4 Discussion

This study aimed to map the evidence on AYAs' healthcare autonomy in the US. AYAs included in this study had several health conditions, including cancer, chronic conditions, sexual and reproductive health issues, mental health, and other specialty services. Among the literature that provided a conceptual definition of autonomy, most referred to the construct of decision-making, while a few referred to adolescents' control, preferences, and choices. Our findings identify the need to consider adolescents' ability to be engaged in planning and decision-making for their healthcare.

Data analysis revealed several definitions and measures of adolescent autonomy, with most focusing on decision-making, while other concepts, such as confidentiality, privacy, or ethical care issues, were consciously lacking. Considering that these concepts have significant implications on healthcare behaviors, outcomes, management, and adherence to treatment plans, particularly for AYAs, ensuring that researchers assess autonomy holistically while developing a clear, meaningful, and practical definition of autonomy can have a serious impact on quality and access to care for adolescents (24), while also meeting the Healthy People 2030 objectives (58). Furthermore, our analysis revealed that only a few articles used validated tools, such as the deciding about diabetes-related conflict (52), the reproductive autonomy scale, and the control preference scale (33) to measure adolescent autonomy. In other situations, authors complemented autonomy measures with other validated scales, such as a three-item CollaboRATE tool to measure shared decision making (33) and the Family Roles Questionnaire (44). Furthermore, autonomy was contextualized based on the intensity of a situation for the adolescent or in context to the adolescent's health (26, 28). Thus, tools used to operationalize autonomy should be streamlined to enhance the validity and reliability of constructs to prevent biased measurement and estimates and ensure that patients are adequately represented (59).

In line with previous research, our study found that adolescents prefer to be actively involved in their care alongside their providers and caregivers (60). Although it is expected that AYAs generally prefer to be included in their care, most studies reporting AYA preferences focus on cancer patients, suggesting a gap in the knowledge regarding factors influencing the preferences of AYAs with other health conditions. Nonetheless, age plays a critical role in AYAs' choices and involvement in their care, with younger AYAs' decision-making being deflected to their caregivers. Previous research suggests cognitive development influences AYAs' ability to manage their health (61). While this perspective holds, we advocate for patient-and family-centered care that actively includes the voices of younger AYAs in their care. Effective patient- and family-centered care ensures that young persons are included in every aspect of their care with support from parents/guardians, emphasizing effective and respectful communication (62).

Moreover, designing health systems and public health interventions with and for young people is a hallmark of collaborative care. Thus, policies, health systems, and health communication materials and processes must be AYA-friendly to ensure AYAs understand fundamental knowledge regarding their care (22, 24, 63).

Using an ecological framework, we found that AYAs' sense of autonomy depends on many interrelated limiting and protective factors within the individual, interpersonal, community/institutional, and policy levels. For example, a patient's age, agency, type of disease, disease diagnosis stage or severity, adverse life events (e.g., incarcerations, etc.), family and friend support, sociocultural norms, ethical and legal issues, and broad policy factors substantially facilitated or impeded AYAs' ability to engage in autonomous care. Thus, our findings highlight the importance of understanding the nuances impacting autonomous care and for interventions to adopt a socioecological or system approach, identifying key areas to leverage to meet the specific needs of the AYAs.

### 4.1 Implications for practice and policy

The findings of this study have serious implications for young people's health care access and outcomes. Firstly, health systems need to be aligned with and center the unique needs of young people in the care delivery process. Developing tools, such as the Confident and Total Teen (64), that enhance young people's agency, confidence, and autonomy significantly impacts adolescent health. Ensuring that health professionals are adequately trained to communicate with AYAs effectively can enhance a sense of agency. Healthcare organizations can play a role by designing policies that allow for the sharing of confidential time between providers and adolescent patients, allowing them the space to reflect on healthcare options and opportunities. Furthermore, future research aimed at understanding adolescents' needs as they relate to their care may offer a starting point for ensuring patient autonomy, leading to better health outcomes and stronger relationships with their providers.

Although a few validated tools and instruments operationalize autonomous care, this study demonstrates a need for streamlined tools that incorporate other aspects of autonomy, such as confidentiality or privacy, or other adjacent factors, such as communicating with providers, navigating the healthcare system, and the ability to manage their health independently, that can affect autonomous care. Health researchers, policy experts, and relevant partners should work towards developing valid and reliable tools to evaluate the different aspects of autonomous care better. Moreover, these tools can be used to understand the AYAs' readiness for healthcare autonomy, and providers can move from assessment to intervening and positively influencing adolescent healthcare autonomy and agency.

Considering that engaging in autonomous care is affected by interrelated factors within the ecological framework, researchers, healthcare professionals, community organizations, and family members must work collaboratively to support AYAs in their healthcare delivery. Thus, it is crucial to understand adolescent autonomy and its influence on young people's access to quality healthcare. Organizational policies and AYA-friendly legislation in healthcare environments that assist AYAs in recognizing, developing, and using their autonomy skills to be the navigators of their healthcare. In addition, family-based healthcare protocols and interventions may help reduce tension between caregivers, providers, and adolescents. Research on family-based interventions has been shown to increase shared making, improve the quality of the patient-provider relationship, improve adherence to treatment and therapy, and achieve better outcomes overall (65, 66). Since legislative and health system factors greatly influence autonomous care. Our study questions the stringent policies and systems that divorce the sociopolitical context that can impede or facilitate AYAs' autonomous care. Therefore, AYAs' autonomy requires that they are addressed holistically through a systems-level lens (24).

### 4.2 Limitations

Our study substantially contributes to the literature to advance young people's health care access and outcomes. However, several limitations must be acknowledged. First, we only included articles published in the English Language, which reflects the practices in the US, and may not be generalizable outside of the US. However, the result may still apply to other regions, given that the need for autonomy is universal across all subpopulations and a human right issue. Second, despite our best efforts, it is likely that we may not have represented the broad scope of scientific literature on AYAs' autonomy. However, we systematically screened the articles and conducted an updated literature search to ensure we included a substantial percentage of AYAs, which is likely not to impact the inferences drawn. Despite this limitation, our study expands the body of knowledge on AYAs' autonomous care and brings to the fore the need for future research in this area.

## 5 Conclusions

The focus of this study was to understand AYAs' autonomous care and decision-making in the healthcare setting in the US. Much literature is centered on decision-making, focusing on knowledge at the expense of other salient factors, such as confidentiality, privacy, or ethical care issues. AYAs’ preferences, barriers, and facilitators of autonomous care are influenced by multiple factors that suggest that improving autonomous care requires a holistic approach that recognizes the complexities AYAs encounter in seeking and utilizing healthcare.
